# PD15 Cost Effectiveness Of The Wolbachia Method For Dengue Control In Brazil

**DOI:** 10.1017/S0266462324002836

**Published:** 2025-01-07

**Authors:** Ivan Ricardo Zimmermann, Ricardo Fernandes, Márcia Gisele da Costa, Márcia Pinto, Henry Maia Peixoto

## Abstract

**Introduction:**

Dengue virus is a significant public health threat in Brazil. It is transmitted by *Aedes aegypti* mosquitoes and causes severe symptoms such as hemorrhagic fever and dengue shock syndrome. This study explored the economic evaluation of Wolbachia mosquito replacement as a promising dengue virus control strategy.

**Methods:**

The model considered Wolbachia mosquito replacement in seven Brazilian cities: Belo Horizonte, Campo Grande, Fortaleza, Goiânia, Manaus, Niterói, and São Paulo. A mathematical microsimulation model tracked 23 million residents over 20 years and considered transitions between five health states (susceptible, asymptomatic, ambulatory, hospitalized, and death). The current dengue control strategy and the incorporation of Wolbachia mosquito replacement were analyzed from both the health sector (public and private) and the Unified Health System perspectives. Direct costs included local dengue control program resources, Wolbachia replacement implementation, and care of patients with dengue virus. The primary outcome was disability-adjusted life-years (DALYs) averted. Sensitivity analyses were also performed.

**Results:**

The model projected 1,762,688 dengue cases over 20 years without Wolbachia replacement. Implementing Wolbachia replacement would prevent at least 1,295,566 cases, demonstrating a high benefit in all simulated cities. Except for Manaus and São Paulo, Wolbachia replacement was dominant (lower costs and higher effectiveness) over current control strategies. Nevertheless, estimated incremental cost-effectiveness ratios for Manaus and São Paulo, which ranged from BRL1,747.11 to BRL5,072.21 (USD309.51 to USD898.56), were well below the Brazilian cost-effectiveness threshold of BRL120,000 (USD21,258.50) for neglected diseases. Furthermore, all incremental net monetary benefit values remained positive for both scenarios in all cities—from BRL41.37 to BRL1,852.42 (USD7.33 to USD328.16).

**Conclusions:**

Wolbachia replacement is a highly cost-effective option in the Brazilian context, aligning with previous international and diverse perspective studies. Sensitivity analysis and alternative scenarios confirmed the robustness of the findings.

